# Transcriptome profiling of two super hybrid rice provides insights into the genetic basis of heterosis

**DOI:** 10.1186/s12870-022-03697-4

**Published:** 2022-06-30

**Authors:** Jun Fu, Yilin Zhang, Tianze Yan, Yanfeng Li, Nan Jiang, Yanbiao Zhou, Qunfeng Zhou, Peng Qin, Chenjian Fu, Haiyan Lin, Jing Zhong, Xue Han, Zechuan Lin, Fei Wang, Hang He, Kai Wang, Yuanzhu Yang

**Affiliations:** 1 Key Laboratory of Southern Rice Innovation & Improvement, Ministry of Agriculture and Rural Affairs, Hunan Engineering Laboratory of Disease and Pest Resistant Rice Breeding, Yuan Longping High-Tech Agriculture Co., Ltd, Changsha, 410128 China; 2 School of Advanced Agriculture Sciences and School of Life Sciences, State Key Laboratory of Protein and Plant Gene Research, Peking-Tsinghua Center for Life Sciences, Peking University, Beijing, 100871 China; 3 College of Plant Science & Technology, Huazhong Agricultural University, Wuhan, 430070 China; 4 Longping Hi-Tech (Sanya) Overseas Seed Industry R&D Co., Ltd, Sanya, 572099 China; 5 State Key Laboratory of Hybrid Rice, Hunan Hybrid Rice Research Center, Changsha, 410125 China

**Keywords:** Heterosis, Transcriptome, Hybrid-rice, Allele-specific expression

## Abstract

**Background:**

Heterosis is a phenomenon that hybrids show superior performance over their parents. The successful utilization of heterosis has greatly improved rice productivity, but the molecular basis of heterosis remains largely unclear.

**Results:**

Here, the transcriptomes of young panicles and leaves of the two widely grown two-line super hybrid rice varieties (Jing-Liang-You-Hua-Zhan (JLYHZ) and Long-Liang-You-Hua-Zhan (LLYHZ)) and their parents were analyzed by RNA-seq. Transcriptome profiling of the hybrids revealed 1,778 ~ 9,404 differentially expressed genes (DEGs) in two tissues, which were identified by comparing with their parents. GO, and KEGG enrichment analysis showed that the pathways significantly enriched in both tissues of two hybrids were all related to yield and resistance, like circadian rhythm (GO:0,007,623), response to water deprivation (GO:0,009,414), and photosynthetic genes (osa00196). Allele-specific expression genes (ASEGs) were also identified in hybrids. The ASEGs were most significantly enriched in ionotropic glutamate receptor signaling pathway, which was hypothesized to be potential amino acid sensors in plants. Moreover, the ASEGs were also differentially expressed between parents. The number of variations in ASEGs is higher than expected, especially for large effect variations. The DEGs and ASEGs are the potential reasons for the formation of heterosis in the two elite super hybrid rice.

**Conclusions:**

Our results provide a comprehensive understanding of the heterosis of two-line super hybrid rice and facilitate the exploitation of heterosis in hybrid rice breeding with high yield heterosis.

**Supplementary Information:**

The online version contains supplementary material available at 10.1186/s12870-022-03697-4.

## Background

Heterosis refers to the phenomenon of the superior performance of a hybrid over its parents in terms of biomass development rates, yield, stress tolerance, and other agronomic traits [1], which is very important for agriculture production. Rice (*Oryza sativa* L.) is a staple food crop for more than half of the world's population. The ability to increase yield potential would be a critical factor in achieving the global rice requirement of 810 million tons by 2025 [2]. Rice is also one of the most important crops which showed the greatest success in heterosis application. Hybrid rice that has a yield advantage of 10%-20% over the conventional varieties was developed and released commercially in the 1970s. The success of hybrid rice has made a great contribution to the self-sufficiency of the food supply in China and world food security. However, the molecular mechanism governing yield heterosis has not been elucidated to date [3].

Since George H. Shull rediscovered heterosis in 1908 [4], three major genetic models have been proposed to explain the mechanisms of heterosis [5, 6]. The first proposed hypothetical genetic mechanism was dominance [5], which states the heterosis caused by the complementation of deleterious recessive alleles [7]. The over-dominance hypothesis attributes heterosis to the superior fitness of heterozygous genotypes over homozygous genotypes at a single locus [4]. The epistasis hypothesis refers to the interaction between alleles from different loci [6]. The current majority of genetic studies on heterosis mainly start from these three hypotheses. The heterosis phenomenon varied with species, traits, and parents [8]. Thus, it is probable that no single genetic mechanism can adequately explain all aspects of that [9, 10]. In rice, dominance [11], over-dominance, and epistasis model [12] have been proposed as underlying mechanisms of the heterosis.

High-throughput sequencing technologies have enabled detailed investigations of the molecular basis of heterosis at the whole genome level [13–15]. With high-throughput sequencing and record the phenotypes of 10,074 F_2_ lines from 17 representative hybrid rice crosses, heterosis-associated loci were identified by GWAS analysis, revealing the genetic mechanisms of heterosis of three different hybrid rice systems [16, 17]. The advent of RNA-Seq has provided an opportunity for transcriptional profiling in heterosis studies [1, 18, 19]. At present, a series of phased progress results have been made in studying the molecular genetic mechanisms of heterosis through transcriptomics. In rice, Wei et al. [20] conducted a comparative analysis of gene expression in seven tissues, including leaves and spikes of super rice Liang-You-Pei-Jiu and its parents. A large number of differentially expressed genes in the F_1_ progeny were significantly higher than that of the parents. The differential gene expression between hybrid and parents can help to clarify the molecular mechanism underlying hybrid heterosis.

Allele-specific expression (ASE) is the phenomenon that only one of the parental alleles was transcribed in the hybrid, which also played an important role in hybrid vigor [21–23]. A total of 3,270 ASE genes were identified in the F_1_ from the cross between ZS97 and MH63 in three tissues under four conditions and be further classified into two categories: 1) ASE genes biased toward one parental allele in all tissues/conditions, and 2) ASE genes biased toward one parental allele in some tissues/conditions while toward the other parental allele in other tissues/conditions. The first type is associated with partial or complete dominance, while the second may lead to over-dominance [24].

Two elite hybrid rice varieties, Jing-Liang-You-Hua-Zhan (JLYHZ) and Long-Liang-You-Hua-Zhan (LLYHZ), were certificated as super rice with high yielding ability by the Ministry of Agriculture and Rural Affairs of the People's Republic of China in 2017 and 2018, respectively. JLYHZ and LLYHZ all showed wide adaptability and got through all of the four state regional trials (the middle and lower reaches of Yangtze River, upper reaches of Yangtze River, South China, and Wuling Mountainous area) with an average increased yield of 6.7% and 7.3%, respectively, and certificated by the national new variety examination and approval committee (NNVEAC). Since getting the first new variety certification in 2015, JLYHZ and LLYHZ had become the top three widely cultivated hybrid rice varieties in China, with annual promotion areas of more than 313,111 and 258,667 hectares, respectively, during 2018–2020. In 2020, JLYHZ and LLYHZ promoted 326,000 and 215,333 hectares, and ranked the first and third most widely cultivated hybrid rice varieties in China, respectively. JLYHZ and LLYHZ were derived from the cross of two thermo-sensitive genic male sterile (TGMS) lines, Jing4155S (J4155S) and Longke638S (LK638S), with the common restorer line Hua-Zhan (HZ), respectively. J4155S and LK638S were two elite TGMS lines developed by Yuan Longping High-Tech Agriculture Co., Ltd. in 2014. In 2021, a total of 40 and 76 hybrid varieties derived from J4155S and LK638S had been developed and certificated by NNVEAC, respectively. The annual promotion area of hybrids of J4155S and LK638S reached more than 2.5 million hectares in 2020. Male line HZ is an elite two and three-line hybrid restorer developed in the 2000s with high combining ability, high disease resistance, high productive tiller number, moderate plant height, and high adaptability for different cultivation regions. So far, at least a total of 158 hybrid varieties have been developed using HZ as the male line. To reveal the underlying mechanism of super hybrid rice, we performed transcriptome sequencing of leaves and panicles of two widely promoted super hybrid rice and their parental lines. Additionally, whole-genome resequencing was also performed on the parents to identify ASE. We identified actively and differentially expressed genes between two hybrids and their parental lines, and analyzed GO enrichment and KEGG enrichment for differential expressed genes. The transcriptome data and resequencing data were used to analyze the genome-wide allele-specific expression genes (ASEGs) of the two hybrids.

## Results

### Gene expression patterns of hybrids and their parents

**Fig. 1:**
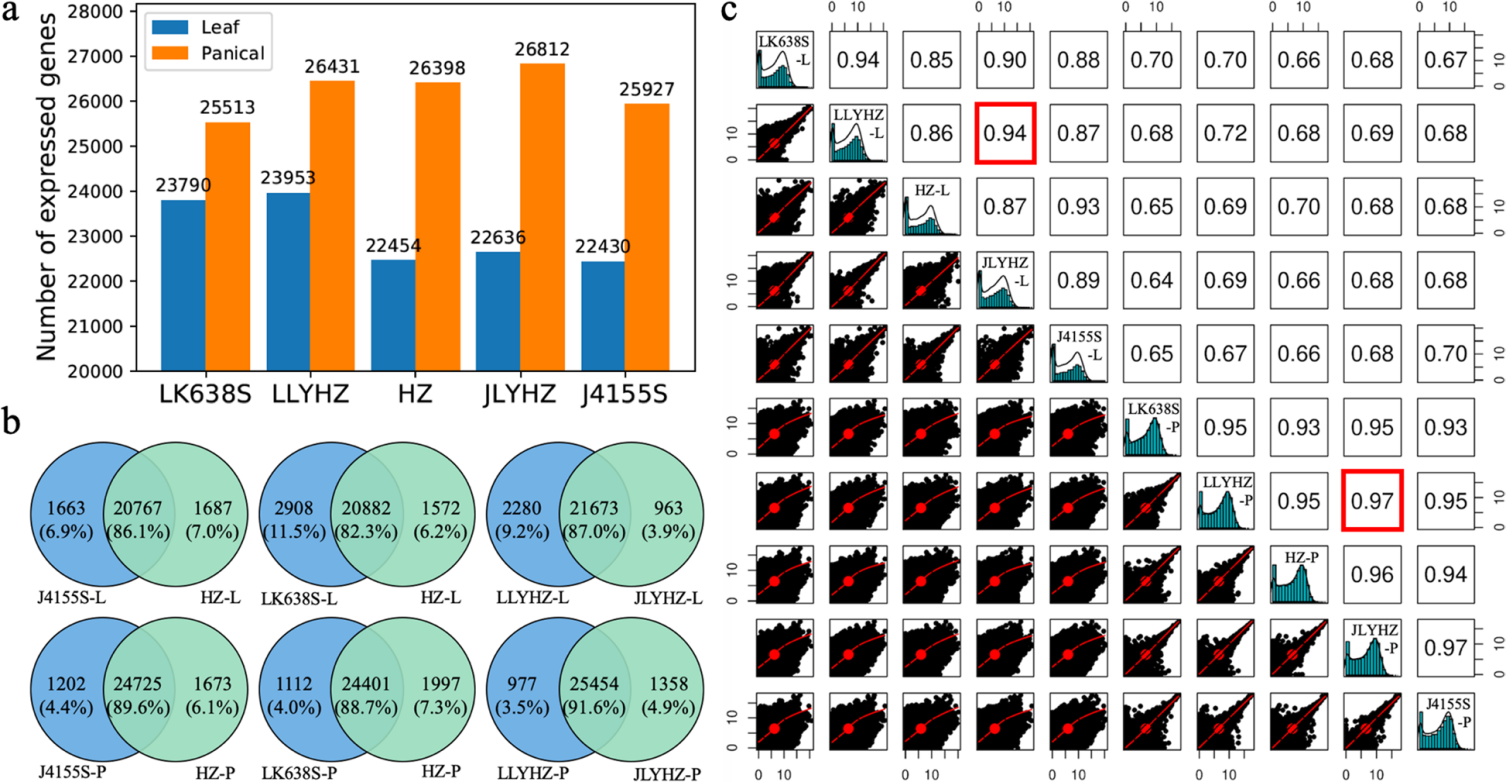
Actively expressed genes and their expression correlation in the hybrids and parents. **a** The number of actively expressed genes in hybrids and their parents. **b** A comparative analysis of actively expressed genes between parents and hybrids and a Venn diagram of co-expressing active genes is obtained. **c** The correlation between the two tissues of the hybrids and their parents is calculated from the expression of all the genes detected in each sample. The correlation coefficients of the two F1 hybrids are marked with a red box

To quantify genome-wide gene expression levels of two super hybrid rice varieties and their parents, young leaves and panicles were collected for RNA-Seq (5 accessions * 2 tissues * 3 biological replicates). A total of ~ 211 Gb high-quality PE150 reads were generated (an average of ~ 7 Gb bases per sample) using the Illumina HiSeq X ten instrument. After removing low-quality reads, sequencing data were mapped to the *japonica* reference genome (IRGSP-1.0) with an average mapping rate of 93.40% (Table S1). Gene expression levels were quantified by normalized read counts. The high correlation of gene expression levels between duplicate samples proves that our data are reliable (Fig. S1). We defined genes covered by at least two reads in at least two biological replications as actively expressed genes. The results showed that the numbers of actively expressed genes in the hybrids were higher than that of the parents in both leaves and panicles (Fig. 1a). Among the five samples, the two hybrids share the most proportion of actively expressed genes than any other pairs in the leaves and the panicles, although LLYHZ and JLYHZ were produced by different crosses (Fig. 1b, S2). In addition, the highest correlation in terms of gene expression patterns was also observed between hybrids (*R*^*2*^ = 0.94 for leaf and 0.97 for panicle) (Fig. 1c). The results implied that similar expression patterns might contribute to the heterosis exhibited in LLYHZ and JLYHZ.

### Differentially expressed genes (DEGs) may play a key role in heterosis

**Fig. 2:**
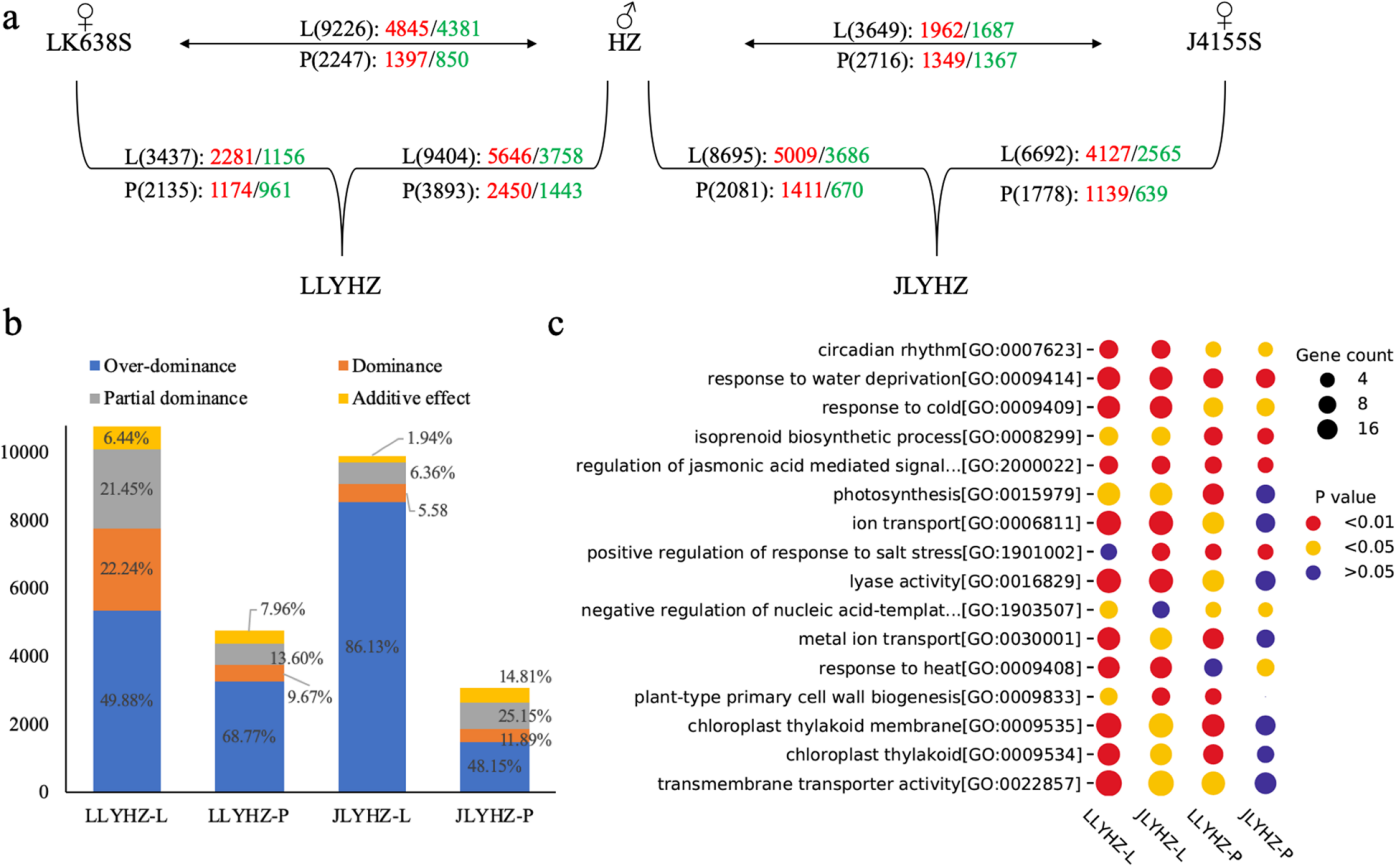
Differentially expressed gene analysis. **a** Statistics of the number of DEGs between hybrids and parents, and between male parent and female parent. L represents leaf, P represents panicle. Numbers in parentheses represent the number of DEGs. Red represents up-regulated DEGs, and green represents down-regulated DEGs. **b** Subdivided patterns in DEGs (Over-dominance, Dominance, Partial dominance, Additive effect) in hybrids. The numbers represent the percentage of different expression patterns, and the different colors represent different expression patterns. **c** The significantly enriched GO terms of DEGs in hybrids and parents. The size of the bubble indicates the number of genes in each module. The color of the bubble indicates a significance level for the GO terms

The numbers of DEGs between hybrids and female parents were less than that between hybrids and the male parent (Fig. 2a, Table S2), which is consistent with the result that the expression patterns of the hybrids were more similar to those of the female parents (Fig. 1c). Compared to the parents, the numbers of up-regulated genes in the hybrids were significantly higher than that of down-regulated genes in both leaves and panicles (Fig. 2a). The numbers of DEGs in leaves were much higher than that in panicles (Fig. 2a). The DEGs between hybrids and parents (DGhp) can be classified into four expression patterns (Fig. S3): over-dominant (the expression level was higher or lower than both parents), dominant (the expression level was comparable to one of the parents), partially dominant (the expression level was between the two parents, but not equal to the median) and additive (the expression level was comparable to the median of the two parents). By analyzing the expression patterns of the DGhp, it was found that most of the DEGs in leaves and panicles were over-dominant for both hybrids (Fig. 2b), especially in leaves of JLYHZ, the proportion of over-dominant differentially expressed genes reached 86.13%.

GO enrichment analysis of the DGhp revealed that the terms significantly enriched in both hybrids and both tissues were circadian rhythm (GO:0,007,623), response to water deprivation (GO:0,009,414), response to cold (GO:0,009,409), regulation of jasmonic acid-mediated signaling pathway (GO:2,000,022, which is associated with biotic and abiotic stress responses [25]) and the isoprenoid biosynthetic process (GO:0,008,299, synthesis of isoprene correlates with respiration, photosynthesis, membrane structure, and growth regulation [26]) (Fig. 2c, Table S3). Significant enrichment in these pathways may account for the wide adaptability of JLYHZ and LLYHZ. KEGG enrichment analysis of the DGhp revealed that the photosynthetic genes (osa00196) were significantly enriched in both tissues of two hybrids. Other significantly enriched pathways were associated with anabolic metabolism, such as the phenylalanine metabolism pathway (osa00360), porphyrin and chlorophyll metabolism pathway (osa00860), carotenoid biosynthesis pathway (osa00906), and thiamine metabolism pathway (osa00730). Notably, genes involved in circadian rhythm (osa04712) and MAPK signaling pathway (osa04016) were also enriched. The MAPK signaling pathway (osa04016) transduces extracellular signals into the cytoplasm or nucleus, which is critical in regulating cell division, differentiation, programmed death, and responses to various stresses [27, 28] (Table S4). These DEGs may play important roles in heterosis formation by regulating growth and development induced by light, which suggested the higher photosynthetic efficiency might be a potential cause of hybrid vigor.

**Fig. 3:**
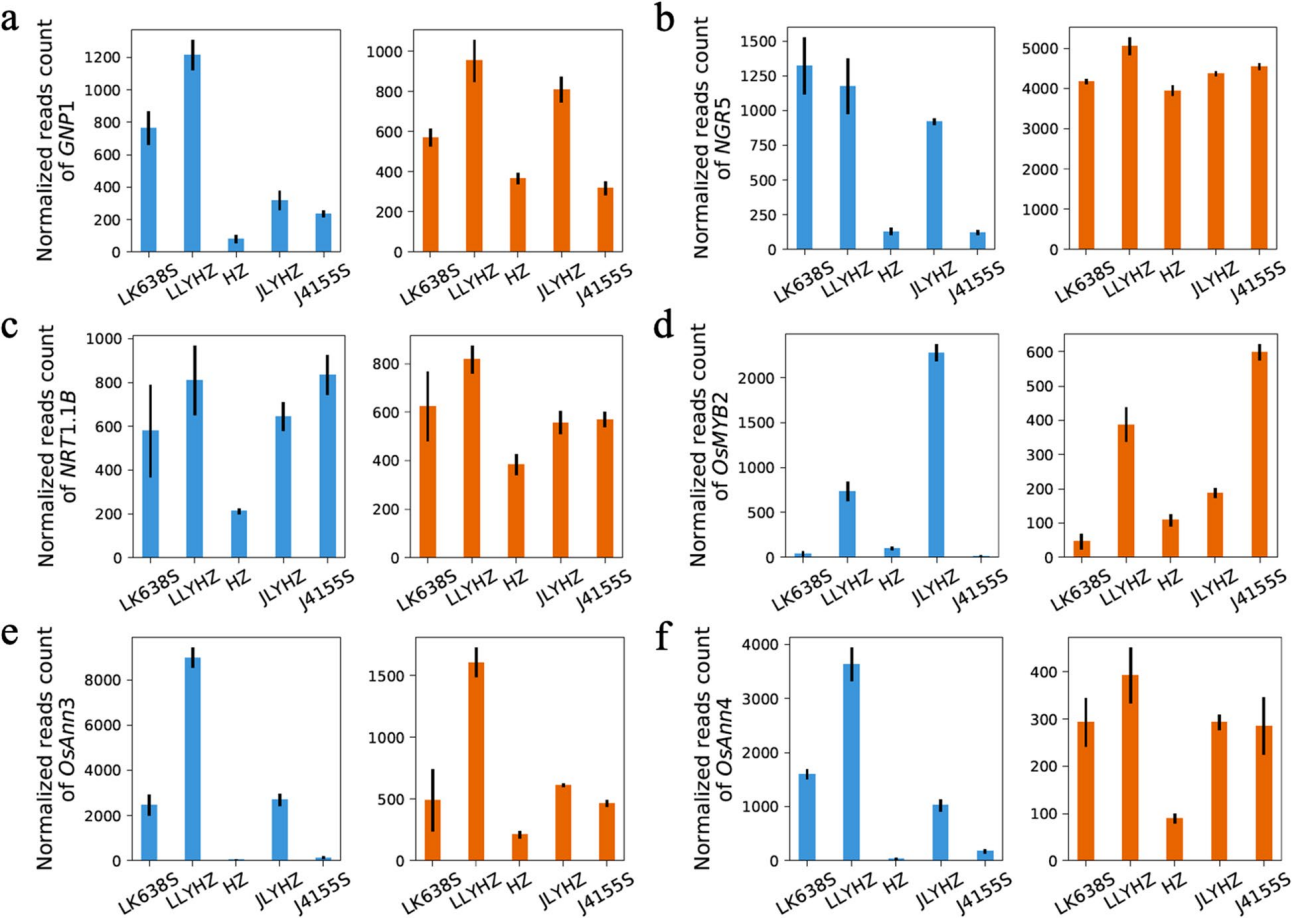
The normalized reads count of some genes that may play a key role in hybrid vigor. The blue bar represents gene expression in leaf, and the orange bar represents gene expression in panicle. **a** *GNP1* gene **b** *NGR5* gene **c** *NRT1.1B* gene **d** *OsMYB2* gene **e** *OsAnn3* gene **f** *OsAnn4* gene

Among the DEGs in the hybrids and their parents, 789 genes were common differentially expressed in both tissues of both hybrids (Fig. S4). Some well-known functional genes involved in yield and resistance were found in the DEGs, which might be responsible for the heterosis performance of JLYHZ and LLYHZ. Such as gibberellin biosynthesis gene *GNP1* involving grain number per panicle development (Fig. 3a), transcription factor gene *NGR5* and nitrate-transporter gene *NRT1.1B* involving nitrogen use efficiency (Fig. 3b, c). In addition, three stress tolerance-related genes, *OsMYB2* [29], *OsAnn3* [30] and *OsAnn4* [31], were over-dominantly up-regulated in the leaves of both LLYHZ and JLYHZ (Fig. 3d, f, e).

### Patterns of allele-specific expression in hybrid rice

**Fig. 4:**
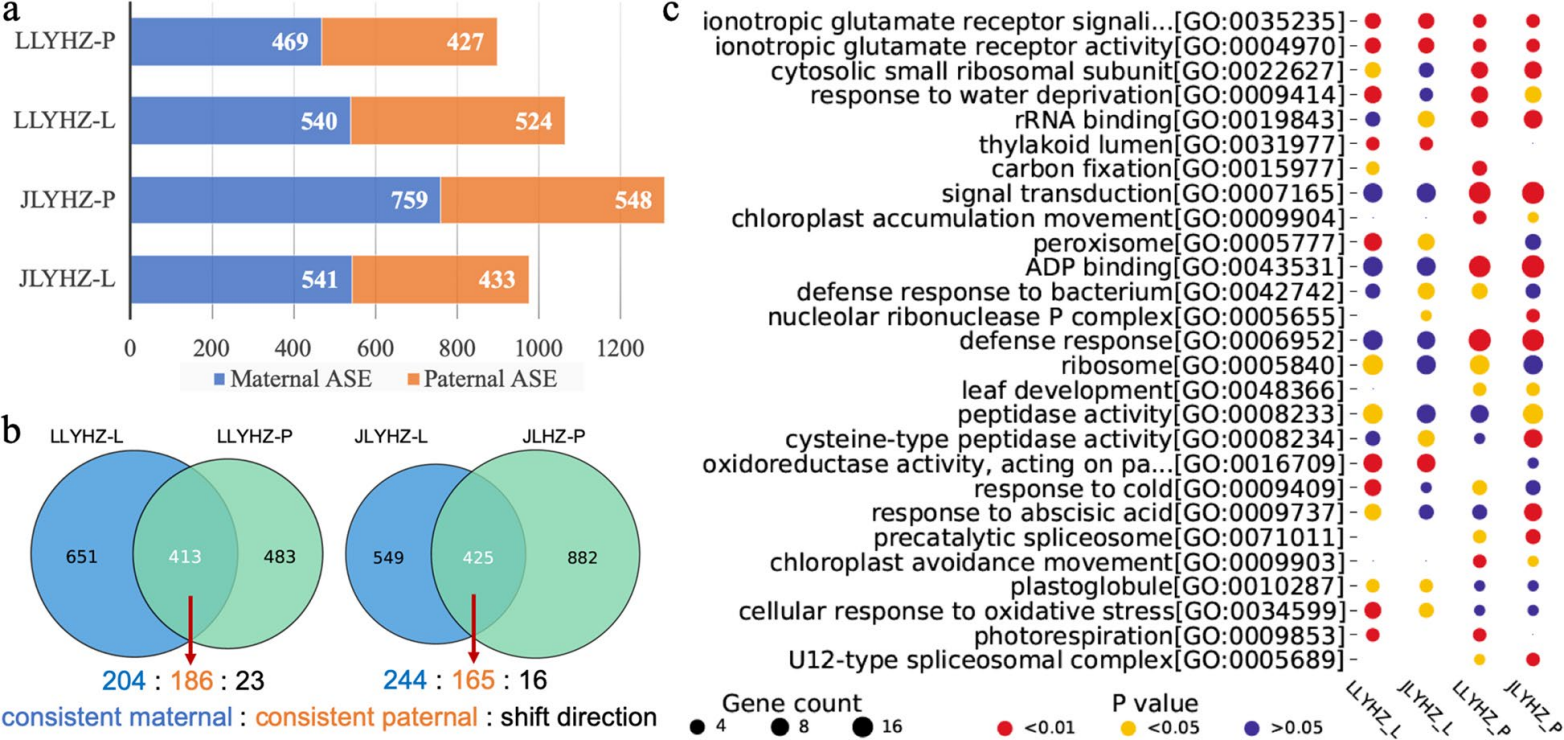
Summary and features of ASEGs. **a** The number of ASE genes in two tissues and two hybrids. Blue color indicates that these ASE genes are biased toward the maternal and orange color indicates that these ASE genes are biased toward the paternal. **b** This Venn diagram represents the number of intersections of ASE genes in two tissues of the same hybrid. The intersection part of ASE genes can be classified into three types: consistent maternal, consistent paternal, and shift direction, and the ratio of these three types is shown. **c** The significantly enriched GO terms of ASE genes in hybrids. The size of the bubble indicates the number of genes in each module. The color of the bubble indicates a significance level for the GO terms

To identify allele-specific expression gene (ASEG) in the hybrids, we performed whole-genome sequencing of the three parents (Table S5). By using the 794,987 detected high-quality homozygous SNPs between LK638S and HZ, and 849,866 between J4155S and HZ as references, a total of 469/427 and 540/524 genes were identified as maternal/paternal ASEG in panicles and leaves of LLYHZ, respectively; 759/548 and 541/433 ASEGs in panicles and leaves for JLYHZ, respectively (Fig. 4a, Table S6). It is noteworthy that the numbers of ASEG from the maternal parent were more than that from the paternal parent in both tissues of both hybrids. By comparing ASEGs in leaves and panicles, we classified ASEGs into three patterns: consistent maternal (specifically expressing maternal genes in both two tissues), consistent paternal (specifically expressing paternal genes in both two tissues), and shift direction (specifically expressing different alleles in different tissues). A total of 390 and 409 consistent ASEGs, and 23 and 16 shift direction ASEGs were identified in LLYHZ and JLYHZ, respectively (Fig. 4b). The phenomenon of specifically constant expression of one of the parental genes in different tissues may be related to the dominant hypothesis of heterosis, and the phenomenon of specific expression of different parental genes in different tissues may be related to the hypothesis of over-dominant of heterosis [24].

By comparing the ASEGs identified in both tissues of the two hybrids with published genes in the funRiceGenes database [32], we found that some of the genes may be responsible for the generation of heterosis in JLYHZ and LLYHZ. As shown in Table S7, the genes associated with heterosis from ASEG may allow the hybrids to acquire increased yield and tolerance. GO enrichment analysis of ASEGs was conducted, and the terms enriched significantly in at least two samples were displayed (Fig. 4c, Table S8). It is worth noting that the glutamate receptor pathway, which was associated with the amino acid perception, was enriched in both hybrid and tissues, significantly. Nitrogen-sensing mechanisms that allow maximizing N use efficiency are essential for the fitness of plants [33]. This is consistent with the high nitrogen utilization characteristics of LLYHZ and JLYHZ. As shown in Table S9, no matter under the conditions of low nitrogen, medium nitrogen, or high nitrogen, LLYHZ and JJYHZ had minimum annual yield variation. Under the conditions of low nitrogen, medium nitrogen, and high nitrogen, LLYHZ increased the yield by 17.3%, 16.5%, and 21.2%, respectively than CK, ranking first among several famous super hybrid rice; JLYHZ increased the yield by 13.1%, 14.6%, and 21.5% respectively than CK, ranking second among several famous super hybrid rice.

### The differentially expressed genes and variations between the parents overrepresented in ASEGs

**Fig. 5:**
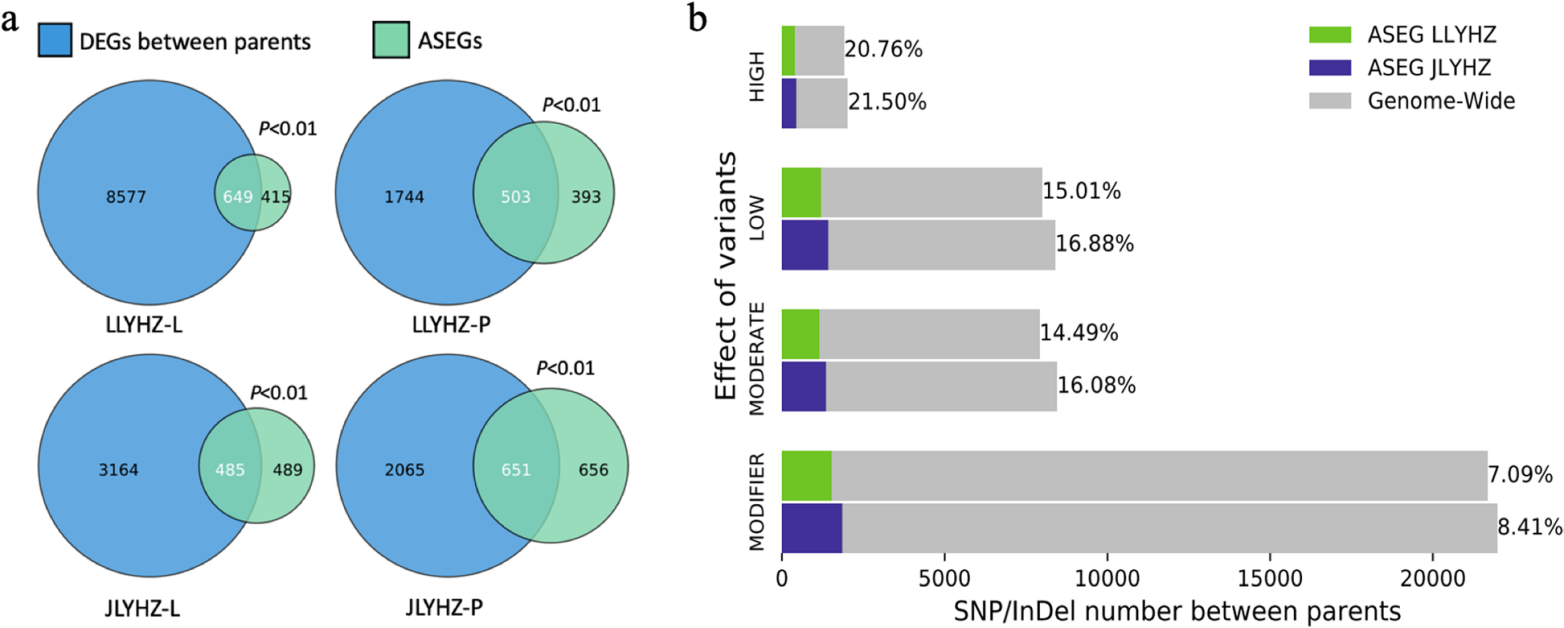
The differentially expressed genes and variations between the parents in ASEGs. **a** The Venn diagrams show the overlaps between parental DEGs and hybrids’ ASEGs. *P* values were obtained by Student’s t-test through 1,000 simulations of random sampling. **b** The SNP/InDel number between parents. The variants were classified into four categories (HIGH, LOW, MODERATE, MODIFIER) according to the effect of the variants on the genes. Green represents the number of variants on the ASEG of LLYHZ, blue represents the number of variants on the ASEG of JLYHZ, and gray represents the number of variants on the whole genome-wide. Percentages represent the proportion of variants carried by ASEGs relative to the whole genome

To determine whether the hybrid ASEGs were also differentially expressed between the parents, we compared the overlaps between parental DEGs and ASEGs with overlaps from two groups of genes sampled randomly. By 1,000 simulations of random sampling, Student's t-test showed that the overlaps were significantly more than expected (Fig. 5a), with a large proportion of ASEGs (49.8 to 61.0%) differentially expressed in the parents. The variations among the parents were further examined and classified into four categories according to their effects on the genes (Fig. 5b). ASEGs contained more variants than the genomic background in all variation types (*p* < 0.01), indicating that most ASEGs were the genes with more variation among the parents. The fraction of the variant types that have greater impacts on protein coding was highest for ASEGs (21.5% and 20.76%), suggesting that some of the ASEGs may have lost their function in one of the parents and a compensatory effect could be achieved by specifically expressing the gene from one of the parents. For example, the *SDS2* gene contains eleven high potential functional mutations, including nine frameshift variants in HZ compared with maternal parents J4155S and LK638S. An *sds2* mutant shows reduced immune responses and enhanced susceptibility to the blast fungus *Magnaporthe oryzae* [34]. *SDS2* shows consistent maternal expression both in JLYHZ and LLYHZ. A similar situation occurs to the *DDF1*, an F-box protein gene that plays pivotal roles in vegetative and reproductive development [35]. HZ carries four missense variants relative to J4155S and LK638S in *DDF1*. The amino acid transporter *OsAAP1* mediates growth and grain yield by regulating neutral amino acids uptake and reallocation [36]. *OsAAP1* contains four missense variants in J4155S compared to HZ and shows consistent paternal expression. Heat shock proteins *OsHsp23.7* play an important role in plant stress tolerance. Overexpression of *OsHsp23.7* enhances drought and salt tolerance in rice [37]. *OsHsp23.7* contains one frameshift and six missense variants in LK638S compared to HZ and shows consistent paternal expression. Collectively, these shreds of evidence attested that allele-specific expression is an important way to achieve heterosis.

## Discussion

In the present study, two elite super hybrid rice, LLYHZ and JLYHZ, and their parents were used to uncover the mechanism of heterosis. Compared with the materials for heterosis research in previous work [19, 24, 38, 39], the hybrids in our study are the latest generation of super hybrid rice in China. LLYHZ and JLYHZ have achieved great success since the first new variety certification was obtained in 2015 and became the top three widely cultivated hybrid rice varieties for three consecutive years (2018–2020) in China. The female parents, LK638S and J4155S, are the leading TGMS lines of Yuan Longping High-Tech Agriculture Co., Ltd., and the male parent, HZ, is the most famous two and three-line hybrid restorer. The genomic and transcriptomic study of LLYHZ, JLYHZ, LK638S, J4155S and HZ will facilitate the timely tracking of the potential genetic mechanism of heterosis of the latest super hybrid rice.

Through the analysis of the transcriptome data of LLYHZ and JLYHZ and their parents, it was found that the hybrids had more actively expressed genes, which may be one of the reasons for heterosis. Both LLYHZ and JLYHZ were more similar to the female parent in expression patterns, which is consistent with the experience of breeders that the female parent has a greater influence on the hybrid than the male parent. Among all DEGs, over-dominantly expressed genes were the largest proportion, and the same phenomenon was observed in other studies [19]. The ratio of over-dominantly expressed genes of JLYHZ in leaves was much higher than that of LLYHZ, which may be the main reason for the significantly low biomass of J4155S, and almost similar biomass of JLYHZ and LLYHZ (Fig. S5). The results indicated that even sterile lines with less biomass could be used to develop elite hybrids with high yield by expressing more over-dominantly expressed genes in hybrids.

The DEGs shared by LLYHZ and JLYHZ might contribute to their high yield, wide adaptability, and stress resistance. For example, *GNP1* was over-dominantly expressed in both tissues of JLYHZ and LLYHZ (Fig. 3a). *GNP1*, which encodes rice *GA20ox1*, is a gibberellin biosynthesis gene. The overexpression of *GNP1* significantly increases grain number per panicle and leads to a higher grain yield [40]. *NGR5* was a crucial element in the GA signaling pathway, which fertilized the utilization of nitrogen in rice. Recent studies have shown that *NGR5* is a positive transcription factor of rice growth and development in response to nitrogen. In the current major high-yielding rice varieties, over-expression of *NGR5* can improve the utilization efficiency of nitrogen fertilizer in rice and maintain its excellent semi-dwarf and high yield characteristics [41]. *NGR5* was over-dominantly and dominantly expressed in the leaves of JLYHZ and LLYHZ, respectively (Fig. 3b). *NRT1.1B* was reported to be involved in nitrogen fertilizer utilization; transferring the *indica NRT1.1B* allele into *japonica* will improve the nitrogen fertilizer utilization efficiency [42]. *NRT1.1B* showed dominant expression in both leaves and panicles for LLYHZ and dominant expression in panicles for JLYHZ (Fig. 3c).

Both LLYHZ and JLYHZ have the characteristics of efficient nitrogen utilization. In general, super rice requires a large amount of N fertilizer input to achieve a high yield [43]. However, for LLYHZ and JLYHZ, they showed stable and high yields no matter under low N fertilizer or high N fertilizer conditions. A field experiment also found that hybrid rice does not necessarily require more N fertilizer to achieve a higher yield than inbred rice [44]. Some genes related to nitrogen utilization, such as *NGR5* and *NRT1.1B*, were identified in common DEGs. The expression levels of *NGR5* and *NRT1.1B* in LLYHZ and JLYHZ were significantly higher than those of their parents, or similar to that of the higher parent (female parent). Moreover, the GO enrichment of DEGs and ASEGs also shows some pathways (GO:0,007,623: circadian rhythm, GO:0,009,414: response to water deprivation, GO:0,035,235: ionotropic glutamate receptor signaling pathway, GO:0,004,970: ionotropic glutamate receptor activity) associated with nitrogen utilization (Fig. 2b, 4c). These may explain in part why LLYHZ and JLYHZ have favorable nitrogen-efficient and yield heterosis.

ASEGs were classified into two major patterns in our study: inconsistent ASEGs (including direction-shifting ASEGs) and consistent ASEGs. The consistent ASE may cause by the fact that one of the parental alleles is functional while the other allele is nonfunctional, like *SDS2*, *DDF1*, *OsAAP1*, and *OsHsp23.7* mentioned in the results. This hypothesis can be proved by the fact that the ASEGs were differentially expressed between the parents, and contained more variations than the background of the whole genome. ASEGs have been reported previously to contain more SNPs [45]. We further found that variants with potential high effects were more overrepresented in ASEGs (Fig. 5b). According to the map of rice quantitative trait nucleotides (QTNs) [46], the effects of variations contained by 63 agronomically important genes were inferred for the three parents. As shown in Table S10, the three parents have many favorable loci related to blast resistance, cold tolerance, more grain per panicle, and other functions. At the same time, if one of the parents contains an inferior allele, the other parent often contains a dominant allele, such as *Pi2*, *OsGSR1*, *TCP19*, et al. All these shreds of evidence suggested that hybrids can select to express favorable genes from one parent to achieve heterosis.

## Conclusions

In conclusion, we provided the transcriptome and annotation of two tissues of the two most widely grown two-line super hybrid rice varieties and their parents. The DEGs and ASEGs between the hybrids and their parents may play an important role in the environmental adaptability and heterosis of the hybrids. However, we only have a small amount of material and cannot accurately answer the relationship between variation and expression. In the future, the mechanism and molecular details of heterosis will be very significant in clarifying and identifying functions of superior alleles in parents that can be used to improve the traits of hybrids.

## Methods

### Plant Materials

Two super rice varieties, Jing-Liang-You-Hua-Zhan (JLYHZ) and Long-Liang-You-Hua-Zhan (LLYHZ), and their female parents Jing4155S (J4155), Longke638S (LK638), and their common male parent HuaZhan (HZ) were used as plant materials in this study. All materials were grown at the Guanshan experimental station of Yuan Longping High-Tech Agriculture Co., Ltd. in the summer season of 2016. At the booting stage, the young panicles and leaves were collected and stored at ultra-low temperature (-80 °C) for RNA sequencing (RNA-Seq) and whole-genome sequencing. Each sample had three biological replications for RNA-Seq.

### RNA library preparation and sequencing

Total RNA was extracted from rice panicles and leaf using Trizol reagent (Invitrogen, CA, USA) and purified using an RNeasy Plant Mini Kit (Qiagen, CA, USA) according to the manufacturer's instructions. RNA degradation and contamination were checked by 1% agarose gel electrophoresis. The quality and integrity of RNA were assessed using an Agilent Bioanalyzer 2100 system (Agilent, CA, USA); RNA Integrity Number (RIN) values were greater than 8.5 for all samples. After total RNA extraction, mRNA was enriched by Oligo (dT) beads. Sequencing libraries were prepared using an Illumina TruSeq RNA Library Preparation Kit (Illumina, CA, USA) as per the manufacturer's protocol and sequenced on an Illumina HiSeq X ten platform, and 150 bp paired-end reads were generated.

### Transcriptome data analysis

After removing low-quality reads with > 5 bases aligned to the adapter sequence or > 50% bases having Phred quality < 20 or contained ≥ 10% unidentified nucleotides, high-quality reads were mapped to the Nipponbare reference (IRGSP-1.0, Ensembl Release 41) genome using HISAT2 [47]. Reads mapped to the rRNA region were removed. Stringtie [48] was used to conduct reference-guided transcript assembly, and the read counts for each gene were measured by Ballgown [49]. DESeq2 [50] was used to test the differential gene expression. FDR and log_2_ values of fold change were calculated. Genes that exhibited an FDR ≤ 0.01 and an estimated absolute log_2_ (FC) ≥ 1 were determined to be significantly differentially expressed genes (DEGs). For each DEG between hybrid and its parents (DGhp), the normalized read counts of two parents and hybrid were denoted as p1, p2, and f1. The additive and dominance genetic effects can be calculated as [a] =|p1-p2|/2 and [d] = f1–(p1 + p2)/2, respectively. According to the value of Hp (= [d]/[a]), we considered that these genes belonged to partial dominance (− 0.8 < Hp ≤  − 0.2 or 0.2 < Hp ≤ 0.8), over-dominance (Hp ≤  − 1.2 or Hp > 1.2), dominance (− 1.2 < Hp ≤  − 0.8 or 0.8 < Hp ≤ 1.2) and additive effect (− 0.2 < Hp ≤ 0.2) [19].

### DNA library preparation and sequencing

Genomic DNA was extracted from young leaf samples using Plant DNA Mini Kits (Aidlab Biotech, China). 1.0 μg of high-quality DNA per sample was used to prepare the libraries. Sequencing libraries were generated using a Truseq Nano DNA HT Sample Preparation Kit (Illumina, USA) following the manufacturer's recommendations; index codes were added to each sample. The insert size of each library was ~ 350 bp. The quality and quantity of libraries were analyzed using an Agilent 2100 Bioanalyzer instrument and qPCR. Whole-genome paired-end reads were generated using Illumina X ten platforms.

### Whole genome re-sequencing and variant calling

The raw paired-end reads generated using Illumina X ten platforms were mapped to the Nipponbare reference genome using BWA [51] directly. PCR or optical duplicates were marked using Picard [52]. We performed SNP and short InDel calling using a HaplotypeCaller approach as implemented in the software GATK3.8 [53]. To remove the potential false positive, SNPs with QUAL < 30.0 or QD < 2.0 or SOR > 3.0 or FS > 60.0 or MQ < 40.0 or MQRankSum < 12.5 or ReadPosRankSum < 8.0 and InDel with QUAL < 30.0 or QD < 2.0 or FS > 200.0 or MQ < 40.0 or ReadPosRankSum < 20.0 were filtered. Gene-based SNP and InDel annotation was performed using SnpEff [54].

### Allele-specific expression analysis

High-quality transcript reads were mapped to the Nipponbare reference genome using STAR [55]. The high-quality homozygous SNPs between parents were used to phase hybrids’ mapped reads. Gene level haplotypic counts were generated using phASER [56]. For testing for allele-specific expression (ASE), allelic counts for each gene were fitted with a negative binomial generalized linear model implemented in the DESeq2 [50] package. The genes with an adjusted P-value ≤ 0.05 were considered as allelic-specific expressions.

### GO and KEGG enrichment

R package ClusterProfiler [57] was used to perform GO and KEGG enrichment analysis. The GO background was acquired from Ensembl BioMart. The Nipponbare (osa) pathway from KEGG was used as background for KEGG enrichment analysis [58].

## Supplementary Information

**Additional file 1: Fig. S1.** The correlation coefficients of all expressed genes between pairs of replicates for each accession. **Fig. S2.** A comparative analysis of actively expressed genes between parents and hybrids in two tissues, and a Venn diagram of co-expressing active genes is obtained. L represents the leaf. P represents the panicle. The numbers represent the number of actively expressed genes. **Fig. S3.** Schematic diagram for the four expression patterns: over-dominant, dominant, partially dominant, and additive. **Fig. S4.** The overlap of DGEs between the hybrids and the parents is shown in a Venn diagram. L represents leaf, P represents panicle. The numbers represent the number of DEGs. **Fig. S5.** The biomass comparison of the hybrids and their female parents. From left to right are J4155S, LK638S, JLYHZ, and LLYHZ.

**Additional file 2: Table S1.** QC and mapping summary of RNA-Seq. **Table S2.** All DEGs between hybrids and parents in both tissues. **Table S3.** GO enrichment analysis for differentially expressed genes in hybrids and parents. **Table S4.** KEGG enrichment analysis for differentially expressed genes in hybrids and parents. **Table S5.** QC and mapping summary of whole genome sequencing. **Table S6.** All ASEGs in both hybrids. **Table S7.** Important ASEGs related to agronomic trait. **Table S8.** GO enrichment analysis for allele-specific gene expression in hybrids. **Table S9.** Yield of several famous super-hybrid rice under different nitrogen treatments. **Table S10.** Types of genes for some important agronomic traits carried by parents.

## Data Availability

The RNA-Seq and WGS data can be downloaded from the GenBank under the project ID PRJNA766708.

## Abbreviations

GO: Gene Ontology
KEGG: Kyoto Encyclopedia of Genes and Genomes
DEG: Differentially Expressed Gene
ASEG: Allele-specific Expression Gene
NNVEAC: The National New Variety Examination and Approval Committee
GWAS: Genome-wide Association Study
RNA-Seq: RNA sequencing
