# Supplementary material for: Transcriptome profiling of two super hybrid rice provides insights into the genetic basis of heterosis
Source: BMC Plant Biol. 2022 Jun 30;22:314. doi: 10.1186/s12870-022-03697-4 (PMC9245205; doi:10.1186/s12870-022-03697-4)
Supplement: Supplementary file 1 — Additional file 1: Fig. S1. The correlation coefficients of all expressed genes between pairs of replicates for each accession. Fig. S2. A comparative analysis of actively expressed genes between parents and hybrids in two tissues, and a Venn diagram of co-expressing active genes is obtained. L represents the leaf. P represents the panicle. The numbers represent the number of actively expressed genes. Fig. S3. Schematic diagram for the four expression patterns: over-dominant, dominant, partially dominant, and additive. Fig. S4. The overlap of DGEs between the hybrids and the parents is shown in a Venn diagram. L represents leaf, P represents panicle. The numbers represent the number of DEGs. Fig. S5. The biomass comparison of the hybrids and their female parents. From left to right are J4155S, LK638S, JLYHZ, and LLYHZ. [file 12870_2022_3697_MOESM1_ESM.docx]

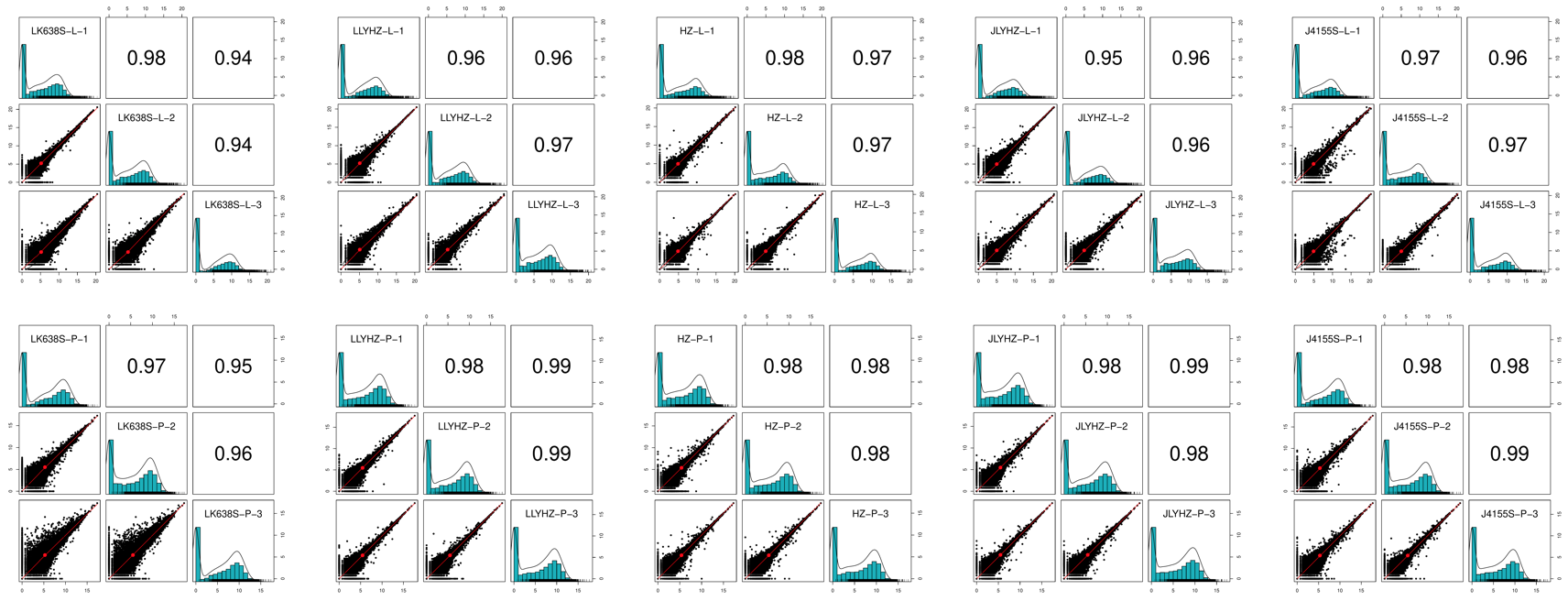


**Fig. S1. The correlation coefficients of all expressed genes between pairs of replicates for each accession.**

**
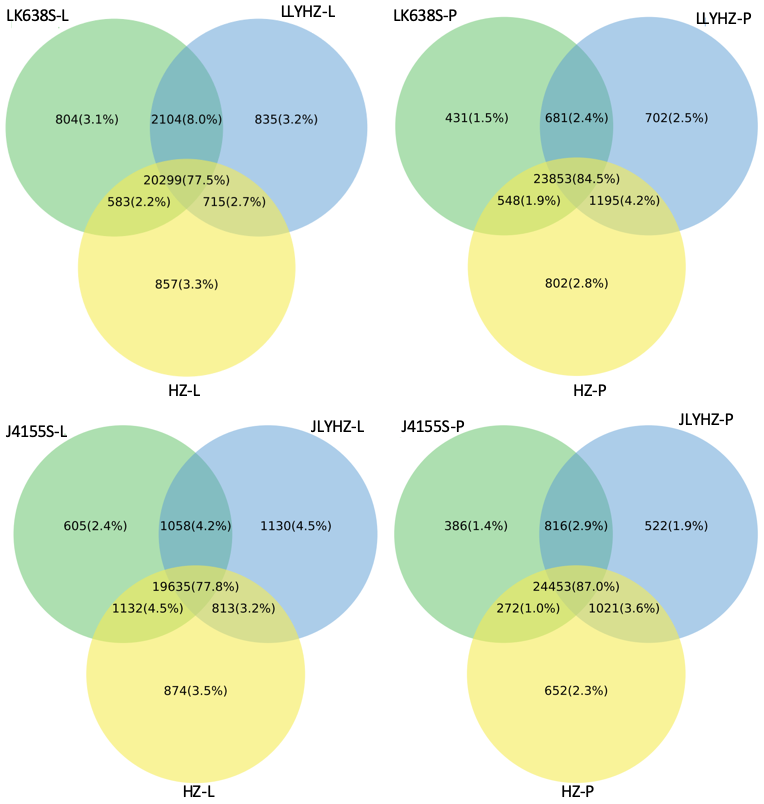
**

**Fig. S2 A comparative analysis of actively expressed genes between parents and hybrids in two tissues, and a Venn diagram of co-expressing active genes is obtained. L represents the leaf. P represents the panicle. The numbers represent the number of actively expressed genes.**

**
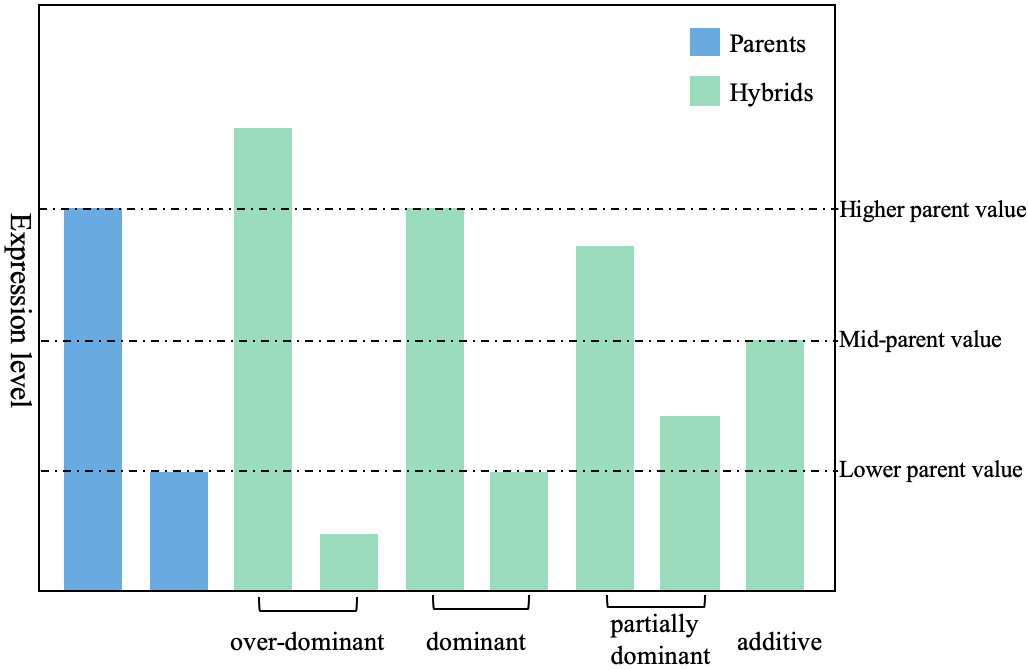
**

**Fig. S3 Schematic diagram for the four expression patterns: over-dominant, dominant, partially dominant, and additive.**


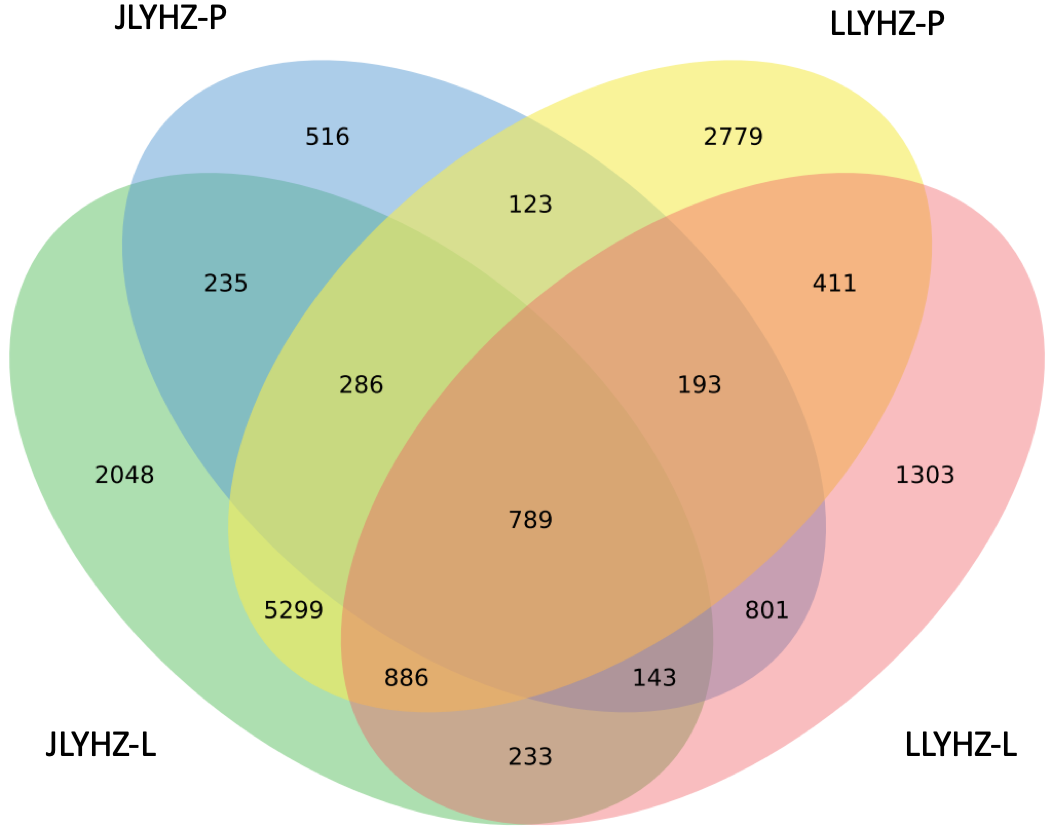


**Fig. S4 The overlap of DGEs between the hybrids and the parents is shown in a Venn diagram. L represents leaf, P represents panicle. The numbers represent the number of DEGs.**

**
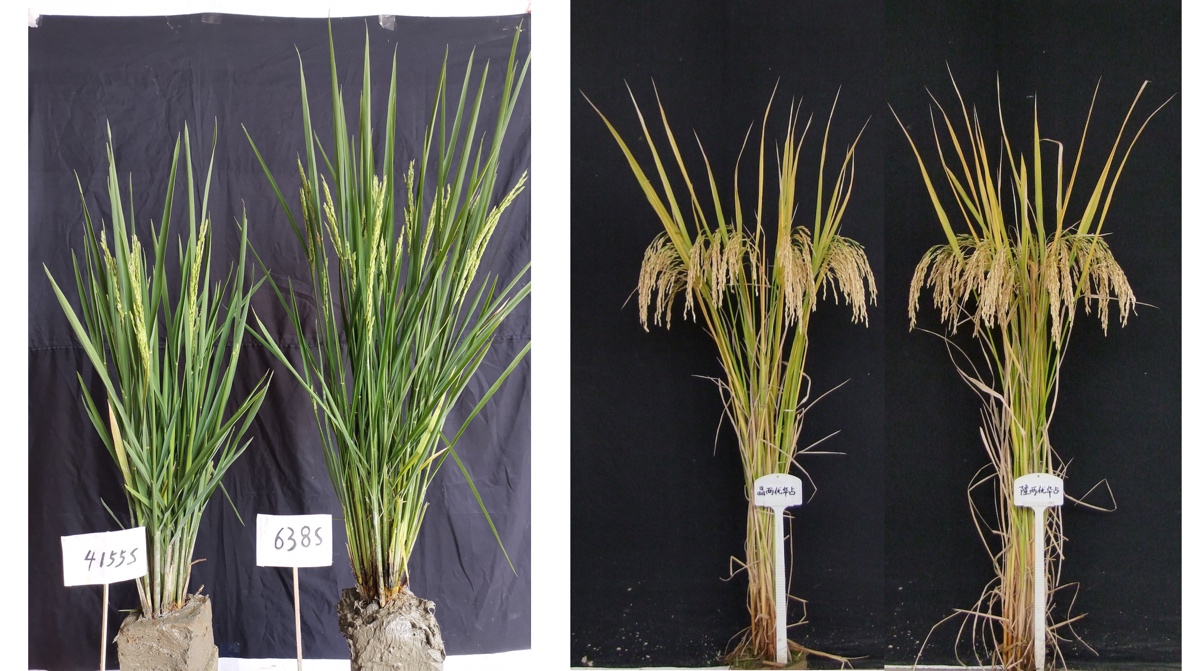
**

**Fig. S5 The biomass comparison of the hybrids and their female parents. From left to right are J4155S, LK638S, JLYHZ, and LLYHZ.**
